# The Discovery and Development of KH607 as a Treatment for Postpartum Depression(PPD)and Major Depressive Disorder (MDD)

**DOI:** 10.1192/j.eurpsy.2025.1382

**Published:** 2025-08-26

**Authors:** J. Wu, P. Chen, L. Song, K. Mu

**Affiliations:** 1 Kanghong Pharmaceutical Group Co., Ltd., Chengdu, China

## Abstract

**Introduction:**

Depression is a serious CNS-related disease that may require long-term treatment. Postpartum depression is a critical medical condition that affects the mother, as well as has a short and long-term impact on the growth of the infant. There is an urgent medical need to develop new treatments for these diseases. GABAA receptor is a cell membrane receptor in the brain that responds to the neurotransmitter gamma-aminobutyric acid (GABA). It is a ligand-gated ion channel and help maintain the balance between excitation and inhibition in the central nervous system, by reducing the likelihood of neuronal firing. It is proven that positive allosteric modulation of GABAA receptor has the potential to treat postpartum depression and major depression disorder.

**Objectives:**

To discover a pre-clinical compound that meets our targeted product profile, i.e., good potency, orally bioavailable, in vivo efficacy in animal disease models, a good safety profile, etc. The compound was to be advanced into clinical development.

**Methods:**

With the help of CADD and AIDD, we have discovered lead molecules that bound to GABAA receptor. Structural optimization of these molecules led to the discovery of a potent GABAA receptor positive allosteric modulator as our pre-clinical candidate (PCC) compound with excellent pharmacokinetic properties and efficacy in a variety of animal models. Safety studies showed that the compound has a good safety profile.

**Results:**

We have discovered a positive allosteric GABAA modulator, KH607, with high potency against all three subtypes of GABAA receptors, especially the α5β3γ2 subtype. PK studies showed that the compound had high oral bioavailability in mice, rats, dogs, and monkeys, ranging from 60% to 125%. KH607 effectively penetrates the blood-brain barrier. In anxiety and depression animal modes, KH607 showed anti-anxiety and antidepressant efficacy through oral administration at a dosage of 0.3 mg/Kg to 1.0 mg/Kg. The phase 1 clinical trial up to 40 mg qd dosage orally in human showed that KH607 was well-tolerated with no severe adverse events (AEs) and low discontinuation rate. The PK result demonstrated good pharmacokinetic characteristics and a linear correlation between dosage and exposure.

**Conclusions:**

PPD and MDD are serious CNS-related diseases that need new treatments. Our newly discovered compound, KH607 is a potent GABAA receptor positive allosteric modulator. Pre-clinical studies showed significant efficacy to improve anxiety and depression-like behaviors in a variety of animal models. The compound has an acceptable safety profile for the development as an anti-depressive drug. Therefore, it was advanced to human clinical studies. A phase 1 clinical trial in human was completed. Phase II clinical studies are currently ongoing. We expect that the compound to be developed as a new treatment to address the unmet medical needs for PPD and MDD patients.

**Disclosure of Interest:**

None Declared

